# Modernistic and Emerging Developments of Nanotechnology in Glioblastoma-Targeted Theranostic Applications

**DOI:** 10.3390/ijms23031641

**Published:** 2022-01-31

**Authors:** Buddolla Anantha Lakshmi, Young-Joon Kim

**Affiliations:** Department of Electronic Engineering, Gachon University, Seongnam-Daero 1342, Incheon 13120, Korea; mamathab72@gmail.com

**Keywords:** glioblastoma, blood–brain barrier, nanotechnology, theranostics

## Abstract

Brain tumors such as glioblastoma are typically associated with an unstoppable cell proliferation with aggressive infiltration behavior and a shortened life span. Though treatment options such as chemotherapy and radiotherapy are available in combating glioblastoma, satisfactory therapeutics are still not available due to the high impermeability of the blood–brain barrier. To address these concerns, recently, multifarious theranostics based on nanotechnology have been developed, which can deal with diagnosis and therapy together. The multifunctional nanomaterials find a strategic path against glioblastoma by adjoining novel thermal and magnetic therapy approaches. Their convenient combination of specific features such as real-time tracking, in-depth tissue penetration, drug-loading capacity, and contrasting performance is of great demand in the clinical investigation of glioblastoma. The potential benefits of nanomaterials including specificity, surface tunability, biodegradability, non-toxicity, ligand functionalization, and near-infrared (NIR) and photoacoustic (PA) imaging are sufficient in developing effective theranostics. This review discusses the recent developments in nanotechnology toward the diagnosis, drug delivery, and therapy regarding glioblastoma.

## 1. Introduction

Glioblastoma is a highly threatening, non-neuronal malignant brain tumor with the lowest survival time of a year to approximately 1.5 years [1]. It mainly emerges in the frontal, parietal, and occipital lobes with vast spreading in the brain parenchyma [2]. Its characteristic features present glioblastoma as a pernicious kind of tumor compared to similar kinds of malignant tumors in other body regions, including diffused growth, vigorous proliferation, substantial edema, distinguished cerebral edema, and multi-invasive capacity into other brain regions [3]. Despite chemotherapy and radiation providing adequate results, surgical resection is becoming a critical procedure because of the infiltration behavior of glioblastoma [4]. Both the complexities of glioblastoma and the physicochemical features of nanotechnology can negatively compromise the targeted delivery options against glioblastoma via either the passive enhanced permeability and retention (EPR) effect or active receptor-mediated delivery [5]. To overcome these challenges, other methods such as argon–helium knife cryotherapy, photodynamic therapy (PDT), and gene therapy have been employed [6,7,8].

The mutations in proteins such as p21, p53, p14, phosphatase, and tensin (PTEN) are responsible for the resistance against chemotherapy and the excessively high infiltration response [9]. Chemotherapy that employs alkylating agents including a plethora of drugs such as temozolomide, carmustine, procarbazine, and lomustine can be therapeutically effective but can suffer from minute aqueous solubility and undesired pharmacokinetics [10]. Unfortunately, less drug retention and high toxicity to surrounding healthy tissues of gliomas limit the clinical success of chemotherapy against glioblastoma [11]. Previously, the diagnosis of glioblastoma had mainly been conducted by using the magnetic resonance imaging (MRI) technique, which often showed inaccurate images. Comparatively, computed tomography (CT), positron emission tomography (PET), functional magnetic resonance imaging (fMRI), ultrasound, and fluorescence-based imaging techniques have been reliable for this purpose [12,13,14,15].

**Figure 1:**
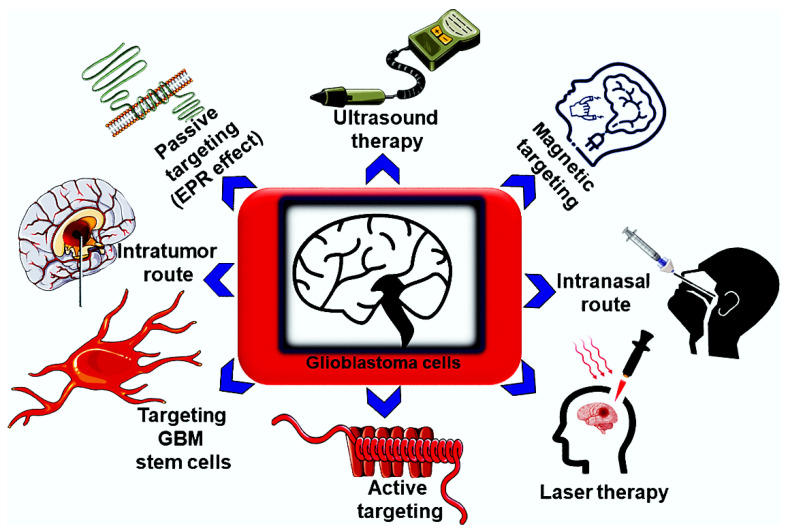
The overview of recent targeting therapies in glioblastoma.

Despite diversified research being in progress for probing therapeutic potential against glioblastoma, a systematic approach based on nanotechnology-based theranostics remains scarce. This might be attributed to the difficult passage of nanomaterials across the blood-brain barrier (BBB), drug resistance, and a profuse infiltrative property [16]. To deal with this context, a multifarious kind of nanomaterial with excellent imaging and therapeutic potential is highly necessary. To investigate the theragnostic behavior of nanomaterials in glioblastoma, a few criteria are considered, which include magnificent optical features (i.e., fluorescence), BBB permeating capacity, adequate biocompatibility, and desirable physicochemical properties. Until now, abundant research has been devoted to theranostics against brain tumors by using a myriad of nanoscale materials [17,18,19,20,21,22,23]. In this regard, nanotechnology-based theranostics can provide a window of opportunities, such as maximum solubility, improved pharmacokinetics, and enhanced targeted therapeutic efficacy. So far, active or passive targeting approaches have been developed for fighting brain tumors. Surprisingly, these tumors have become highly resistant to therapeutics and radiation therapy, which urges the advent of other therapies, such as magnetic, ultrasound, stem-cell-based, intratumoral, and intranasal targeting, etc. (Figure 1). Further, these are extremely adequate for producing antitumor effects in glioblastoma by delivering hyperthermic, magnetic stimuli-based thermolysis, radio-immuno, and photothermal properties. This review presents the latest developments in nanotechnology-based theranostics for exploring glioblastoma.

## 2. Challenges and Strategies of the Nanotechnology against Glioblastoma

**Figure 2:**
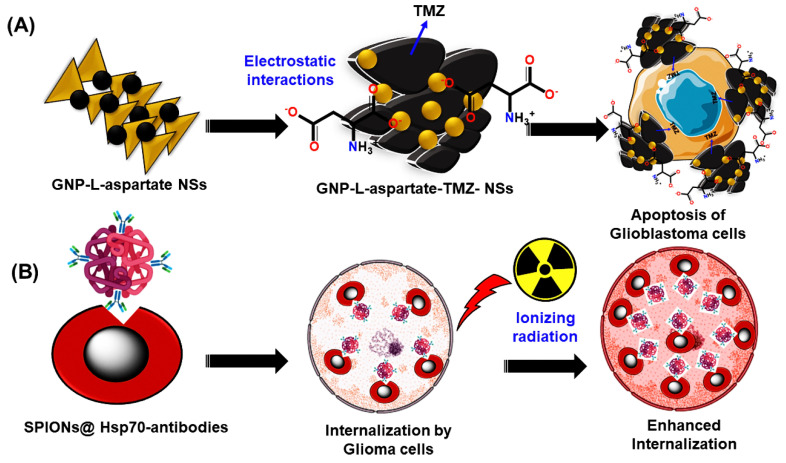
(**A**) The illustration for the prevention of chemotherapy through gold nanoparticle-based L-aspartate-conjugated TMZ nanostructures, and (**B**) the scheme for induced radiosensitization by SPIONs-conjugated Hsp-70 antibodies.

Currently, nanotechnology is associated with different challenges in the treatment of glioblastoma, which mainly includes the structural complexity of BBB and the existence of chemotherapy resistance. To combat these issues, several strategies have been developed by alternating critical modifications in nanotechnology. Already, some traditional therapies (i.e., DNA alkylating or methylating agents) have evidenced a reduced efficacy against malignant glioma cells and have induced chemoresistance. Thus, advanced strategies can overcome these limitations by using the potential combination of conventional therapy and emerging nanotechnology-based technologies. For this purpose, the effective application of very minimal toxic chemotherapy along with nanotechnology-based therapeutics can aid against glioblastoma. Noteworthily, these nanomaterials can cross the BBB and increase the activity of drugs (i.e., temozolomide) with enhanced accumulation and efficiency. For instance, Orza et al. designed gold nanoparticles (AuNPs)-L-aspartate nanostructures, which can conjugate with temozolomide by electrostatic interactions and potentially deal with the recurrent glioblastoma by exhibiting approximately 90% apoptosis as compared to that of temozolomide alone (ca. 42%) [24]. Primarily, this behavior has been believed to be due to physicochemical properties such as the nano size, surface modification, and concentration of nanomaterials, which help in the magnificent internalization capacity against active therapeutic drugs. Figure 2A represents the electrostatic interactions between L-aspartate and AuNPs and shows the apoptosis of glioblastoma by the gold NPs-L-aspartate-TMZ nanostructure.

Resensitization of glioma cells against temozolomide has become possible through preventive autophagy utilizing chloroquine and doxorubicin (DOX). For example, stimuli-responsive AuNPs were reported to target the glioma via their aggregation with asparaginyl endopeptidases such as legumain and increased the “DOX and hydroxychloroquine (HCQ)” accumulation, which greatly contributed to their synergism against cell proliferation in glioblastoma [25]. Nanomaterials can combat radiosensitization by implementing potential strategies such as passive and active targeting processes. Firstly, passive targeting involves the specific accumulation of nanomaterials within the tumorous tissues without affecting healthy tissues. In this context, the EPR effect of the nanomaterials can allow their passage toward the tumor environment rather than non-tumorous tissues, which selectively depends on the size of the nanomaterials [26]. Moreover, prolonged circulation time of nanomaterials can lead to a maximum half-life within the blood, and thereby the chances of prolonged concentrations of nanomaterials are possible. For instance, polyethylene glycol (PEG) coating can be a beneficial phenomenon for extending circulation time as well as achieving increased stability across physiological fluids [27]. Later, the disruption of the BBB is not uncommon in glioblastoma and brain metastasis. Consequently, an excessive permeability of the BBB can be triggered, which obstructs the safer EPR effect through the nanomaterials. In this case, the physicochemical features of the nanomaterials, including lipophilicity, size, and ionization potential, should be properly tunable to provide a successful EPR effect. Unfortunately, passive targeting strategies are still in the budding stage to uncover the potential benefits against glioblastoma and other aggressive tumor types. Hence, active targeting can have better scope due to the facile functionalization by nanomaterials such as ligands (i.e., peptides, antibodies, and specific surface proteins). A few strategies depend on the utility of monoclonal antibodies, which exhibit meritorious selectivity and specificity toward the targeted glioma tissues with an adjustable binding interaction [28]. Shevtsov et al. developed superior theragnostic-based superparamagnetic iron oxide nanoparticles (SPIONs) that are conjugated with the antibodies of a membrane heat shock protein (Hsp70.1). These NPs can enhance their accumulation in glioblastoma cells and thereby act as promising diagnostic agents owing to their magnificent visualization of tumors under magnetic resonance imaging (MRI) [29]. The efficient internalization of SPIONs-conjugated Hsp70 antibodies into the glioma cells is illustrated in Figure 2B.

Significantly, metal-based NPs such as silver (Ag), Au, and gadolinium (Gd) NPs are promising candidates for combating glioblastoma. For instance, citrate-capped Ag NPs show more preferentially magnificent radiosensitizing properties than citrate-capped Au NPs [30]. A reasonable curing rate of about 40% was observed in rats having glioma under 10 Gy radiotherapy using Ag NPs at a concentration of 10/20 μg [31]. In another study, Tamborini and his coworkers developed chlorotoxin-modified Ag NPs using radiation of 2 Gy, which efficiently prevented tumors [32]. This suggested that Ag NPs are potential radiosensitizers against glioblastoma.

## 3. Recent Developments in the Nanotechnology for Investigating Glioblastoma

**Figure 3:**
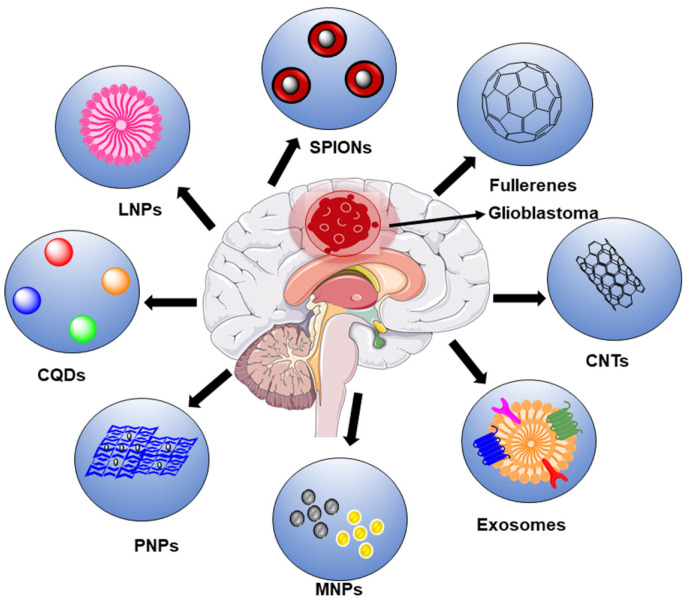
Recently developed nanomaterials in the diagnosis and treatment of glioblastoma.

Several nanomaterials are desirable in investigating the optimal nanoprobe for glioblastoma, including exosomes, carbon-based quantum dots (QDs), graphene oxide (GO), and carbon dots (CDs), and polymer-based, liposome-based, magnetic-based, and metal-based nanomaterials (Figure 3).

Unfortunately, no particular approach is versatile enough to target brain tumors due to their problems in maintaining ideal physicochemical properties. However, the mixed nanotechnology strategy is suitable for obtaining promising results against glioblastoma. To achieve fruitful targeting toward glioblastoma, potential ligands such as peptides, antibodies, and other biomolecules are necessary. Considering the potential surface functionalization of nanomaterials is possible under the desired environments, i.e., known or expected expressions of targeted receptors or enzymes on glioblastoma. In the subsequent subsections, we discuss the advancements in nanotechnology in targeting glioblastoma.

### 3.1. Exosomes

Exosomes are typically phospholipid, bilayered nanoscale secretions (ca. 30–150 nm), which transport drugs, nucleic acids (i.e., DNA, mRNA), and proteins against several tumors. The exosome structure is highly beneficial in avoiding enzyme degradation and prolonging the half-life of the drug and finally aids in the enhanced bioavailability of the drugs [33]. Qi et al. developed blood exosome-derived clusters of superparamagnetic nanoparticles with an excellent in vivo targeting efficiency compared to that of superparamagnetic nanoparticles alone [34]. Again, glioblastoma releases myriad kinds of exosome-like membranous vesicles into the blood, which can play a significant role as promising biomarkers in diagnosing and treating glioblastoma [35]. These exosomes can be absorbed by the healthy cells (i.e., brain endothelial cells) surrounding glioblastoma and result in a massive increase in the production of glioblastoma (i.e., cell proliferation). The expressions of the epidermal growth factor receptor (EGFR) EGFRvIII are remarkably high in the serum samples of glioblastoma patients in contrast to that of healthy individuals. Hence, extensive study of these exosomes may be sufficient to detect the expressions of EGFRvIII [36]. To achieve sufficient anti-tumor potentials against glioma, nanomaterials should be able to cross the BBB and accumulate more time without any biodegradation. Li et al. prepared boron CDs using boron phenylalanine (BPA) and glucose as precursors through hydrothermal heating and coating by exosomes to explore the activity of boron neutron capture therapy (BNCT) against glioma in mice. More importantly, these nanomaterials have shown a more excellent permeability across the BBB than the CDs alone (staying only up to 15 min), and then they shed from the brain [37]. Furthermore, their fluorescence imaging properties are adequate for precisely identifying glioma, which qualifies these materials as “promising theranostics”. Noteworthily, the embryonic stem cells (ESCs) can demonstrate pluripotency and can inhibit the tumor microenvironment through a reprogramming approach where severe malignant tumors can be transformed into minimal malignant tumors [38]. It is already well known that ESCs are huge producers of exosomes, which can develop a plethora of chemotherapeutic nanocarriers against malignant tumors, i.e., glioblastoma [39]. Unfortunately, an intravenous injection of exosomes can accumulate more in the liver than in the brain; this is the bottleneck for exosome delivery. To address these issues, the surface modification approach on the exosomes via using targeting ligands can enhance the retention capacity of exosomes in the brain [40]. For instance, Zhu and his coworkers have developed the cyclo (Arg-Gly-Asp-D-Tyr-Lys) c(RGDyK) peptide and paclitaxel (PTX)-conjugated ESCs-derived exosomes, which exhibit an extreme targeting potential against glioblastoma multiforme (GBM) [41]. These results shed new light on the two aspects, one being the usage of ESCs and the other being the functionalized ligands (such as peptides) for their production and targeting ability toward glioma, respectively. In another study, a combinational drug therapy (TMZ and O6 -benzylguanine (BG)) using exosomes as nanocarriers reduced the chemoresistance of TMZ and showed maximum therapeutic efficiency against glioma [42].

### 3.2. Carbon-Based Nanomaterials

Fullerenes are zero-dimensional (0D) carbon-based nanomaterials that typically exhibit distinct features such as ultra-small size, consistent dispersion, and magnificent reactivity. Fullerenes (C_60_) struggle with solubility issues both in aqueous and organic solvents due to their excessive hydrophobicity, which limits their biomedical applications. To deal with this issue, highly water-soluble and functionalized forms of C_60_ were developed by researchers, which showed excellent results against glioblastoma by inhibiting the growth of the tumors. In particular, the phenylalanine functionality may have adjusted the oxygen (O_2_) metabolism in the glioma cells and exerted neuroprotective benefits [43]. Peng and his coworkers have developed outstanding water-dispersible C_60_ conjugates labeled with ^64^Cu and functionalized with cRGD peptide and 4,7-triazacyclononane-1,4,7-triacetic acid (NOTA) for targeting α_v_β_3_ integrins in glioblastoma [44]. Further, their distribution was visualized in tumor-bearing mice through PET imaging. These results highlight the significance of adequately aqueous-soluble and peptide-conjugated fullerenes in the development of potential imaging and targeting probes in glioblastoma. Unfortunately, uncontrollable toxicity is a significantly considerable parameter for nanomaterials, which should be minimized by thorough modifications in their physicochemical properties. However, the toxicities of non-functionalized fullerenes (C_60_ and C_70_) are higher compared to that of functionalized fullerenes. For instance, Usenko et al. have observed these toxicities in zebra fish embryos, which suggested that hydroxyl-functionalized fullerenes, i.e., C_60_(OH)_24_, comparatively exhibited low toxicities [45]. This is because of a lack of apoptosis cell death by the functionalized fullerenes, whereas non-functionalized fullerenes lead to apoptosis-mediated cell death, thereby causing higher magnitudes of toxicities. Thus, functionalization or surface modification in the fullerenes is a remarkable parameter to control and monitor these toxicities and biodistribution for in vivo settings.

Functionalized carboxyfullerenes (C_70_) such as malonic acid-conjugated fullerenes are also found to be effective due to their antioxidant nature by preventing reactive oxygen species (ROS) that can be used in oxidatively stressed tumors [46]. Metallofullerenes (i.e., Gd_3_N@C_80_) functionalized with hydroxyl and carboxyl moieties (i.e., Gd_3_N@C_80_(OH)_26_(CH_2_CH_2_COOH)_16_) were able to conjugate with the fluorescent label, i.e., 5(6)-carboxytetramethylrhodamine (TAMRA), and peptide, namely interleukin-13 (IL-13), which is a significant receptor on the GBM cells. These gadolinium-based metallofullerenes demonstrated magnificent imaging and targeting abilities against GBM [47]. Meanwhile, the application of radio-fullerenes as nanocarriers for delivering radionuclides by conjugating a targeting ligand (i.e., IL-13 peptide) in radio-immunotherapy (RIT) is also becoming an exciting study for investigating brain tumors. For example, β-emitters and TAMRA-labeled IL-13 peptides conjugated to the radiolanthanide lutetium (^177^Lu) encapsulated C_80_ fullerene cage (^177^Lu_x_Lu_(3-x)_ N@C_80_), which can show potential therapeutic properties toward GBM [48]. Interestingly, these two kinds of fullerenes are good candidates for MRI and X-ray contrast agents, respectively. Moreover, radionuclides such as β-emitters have the potential of ultra-short range of penetration and localized radiation to achieve a therapeutic value in targeted sites.

Graphene and reduced GO nanomaterials still lack hydrophilicity and biocompatibility properties to be promising nanomedicines in anti-tumor applications. To combat this, researchers have been developing multifunctional nanomaterials using polymers (i.e., PEG) and targeting peptides (IL-13) with a mesoporous silica coating on the GO nanosheets. Hence, these are superior, with a combination of chemotherapeutic/photothermal therapy (PTT) and an excellent MRI potential against glioma exhibiting high aqueous solubility, very minimal toxicity, ultimate near-infrared (NIR) absorbance, and a maximum drug loading efficiency [49]. Recently, the integration of PTT and radiotherapy via utilizing a radiosensitizer has been a “valuable therapeutic hotspot” in removing glioma tumors. For instance, Kargar and his coworkers developed poly lactic-*co*-glycolic acid (PLGA)-functionalized nano GO materials loaded by a radiosensitizer, namely 5-iodo-2-deoxyuridine (IUdR). These materials achieved maximum cytotoxicity for U-87MG cells by releasing IUdR (ca. 23.7%) within 5 min [50]. Furthermore, Shirvalilou et al. developed PLGA-coated, IUdR-loaded magnetic nano GO SPIONs for enhancing the effect of the radiosensitizer, which resulted in potential apoptosis by showing elevated expressions of Bax/Bcl-2 in glioma [51]. The nanocomposites made of GO nanosheets and ZnO NPs and modified with Pluronic have shown dual-mode chemotherapy with PTT effects against glioblastoma with increased biocompatibility [52].

Surface modification of graphene quantum dots (GQDs) seems to be a promising approach to obtaining biocompatibility and allows a synergistic effect when used with an anti-tumor drug such as doxorubicin (DOX). This behavior lies in the induction of membrane permeability across the BBB. Perini et al. synthesized three kinds of GQDs; GQDs without functionalization, carboxyl-modified GQDs, and amine-modified GQDs. The synergistic effect of carboxyl-modified GQDs and pristine GQDs with the DOX has shown an adequate glioma reduction as compared to the DOX alone. However, no change in the membrane fluidity was found in the neurons of the cortex in mice embryos [53].

NIR CQDs have been superiorly explored for the imaging of aggressive tumors such as glioma due to their higher penetrating abilities toward biological tissues compared to visible light. Meanwhile, ultrasonic waves can have a minimal scattering effect in the tissues than the photons; thereby, photoacoustic (PA) imaging can enable adequate penetration features compared to optical imaging. Therefore, the dual-mode of the NIR/PA imaging platform can contribute to maximum spatial resolution in the tumor tissues. For instance, S. Li et al. synthesized CQDs from precursors, namely 1,4,5,8-tetraminoanthraquinone (TAAQ) and CA, which displayed the abovementioned dual imaging with higher degrees of specificity toward glioma [54]. These CQDs can load typical chemotherapeutics such as DOX, hydroxycamptothecin, and topotecan hydrochloride (TPTC) via their π–π stacking interactions obtained from the aromatic rings.

Carbon nanotubes (CNTs) are the other outstanding materials, which have displayed imaging, therapeutic, drug-carrier, and targeting potential that can make them suitable to be considered as the leading theranostics in investigating glioblastoma. Unfortunately, their agglomeration in the biological system can result in unwanted reactions, such as incompatibility, inflammation, and thrombosis, etc. Additionally, physicochemical and optical properties including size, length, dispersion, concentration, chemical functionalization, NIR absorption, and Raman spectral features are very crucial in determining their biodistribution and cytotoxicity for cancer research [55]. In this regard, the short-lengthened and agglomerated forms of single-walled CNTs (SWCNTs) can facilitate the selective photoacoustic destruction of glioma under NIR laser irradiation without affecting the nearby tissues [56]. Another research group has developed SWCNT-DNA complexes, which can accumulate and localize inside the glioma cells at very fast rates while maintaining a significant mitochondrial potential and ROS production against glioma [57]. The integration of PTT and molecular targeting has also been a crucial platform for investigating the effects of SWCNTs. Due to their convenient photothermolysis properties, SWCNTs conjugated with CD133 monoclonal antibodies (MoAb) have shown significant cytotoxic effects in glioblastoma under NIR laser irradiation [58]. Moreover, the application of low-frequency and low-strength electrical stimulation of glioma cells could achieve a rapid and maximum penetration capacity of SWCNTs via the cell membrane and induce the effectiveness of SWCNTs against glioma. This facilitated the magnificent amplification of SWCNTs within the cells. Excitingly, it allowed the very minimal usage of drug application via carbon-based nanomaterials and enhanced the endocytosis of CNTs into the glioma [59].

### 3.3. Liposomes

Liposomes are phospholipid bilayered structures consisting of an aqueous core arranged in a variety of unilamellar and multilamellar vesicular forms. These are highly biocompatible and efficiently biodegradable with a suitable entrapment characteristic for hydrophilic and lipophilic drugs. In essence, homeostasis of the brain is significant for escaping the advent of tumors and it can be regulated by the expressions of numerous receptors, including transferrin, insulin-like growth factor, leptin, insulin, and low-density lipoproteins from the brain capillary endothelial cells (BCECs). Therefore, nanotechnology using receptor-mediated transcytosis (RMT) has been encouraged for the designing of functionalized ligands against brain tumors [60]. In this regard, liposomes should bind to the targeted ligand to achieve the desired cellular uptake. For instance, cobalt porphyrin phospholipid (Co-PoP) liposomes were efficiently up-taken by the glioblastoma (U-87) cells due to the strong binding of polyhistidine (his)-tagged RGD peptides to the liposomes [61]. Herein, the binding of a suitable quantity of peptides is a critical factor for maintaining an adequate cellular uptake of NPs into the cells. The other dual ligand (angiopep-2 and A15)-modified cationic liposomes were synthesized to deliver a combination of PTX (a drug) and survivin siRNA against glioblastoma and glioma stem cells [62]. As survivin is a highly expressed marker in glioma cells, its expression is regulated by using these liposomes. This study suggested that these liposomes can enable the differentiation of glial stem cells into non-tumoral stem cells by targeting the CD133 marker in glioma cells.

Moreover, the drug resistance adopted by glioblastoma has also shown limited efficacy by the administration of any single drug through the NPs. For example, paclitaxel-loaded liposomes conjugated with the transferrin (TfR) receptor and RGD peptide have shown higher rates of cellular uptake and have efficiently targeted the αvβ3 integrins expressed by glioblastoma [63]. In another study, DOX-loaded liposomes were developed using dipeptide ligands (^D^CDX) of nicotine acetylcholine receptors (nAChRs) and ligands of integrin receptors c(RGDyK). Due to the prolonged blood circulation times of these ligands, the targeting ability of these liposomes in glioblastoma was efficiently enhanced. Especially, ^D^CDX ligands have shown preferable binding potential with nAChRs, and c(RGDyK) ligands have exerted outstanding stability toward the liposomes because of their cyclization and small peptide sequence. Hence, multiple barriers, such as enzyme degradation, the BBB, and the BBTB (blood-brain tumor barrier), can be overcome and strategic glioblastoma targeting by these liposomes can be attained [64]. Unfortunately, either paclitaxel or DOX are not stand-alone drugs for glioblastoma therapy. In this sense, combined drug therapy will have promising and reliable outputs, particularly to the infiltrative and drug-resistant glioblastoma. For instance, Lam et al. developed transferrin-modified liposome NPs for a dual-drug-targeted therapy for glioblastoma. For this, they loaded the PEGylated liposome NPs with the TMZ and bromodomain inhibitors (i.e., JQ1), which demonstrated the enhanced damage of DNA and apoptosis in the glioblastoma cells [65]. Interestingly, TMZ is already approved by the Food and Drug Administration (FDA) and bromodomain inhibitors have been investigated in clinical trials for glioblastoma treatment. The utilization of bromodomain inhibitors can open a new window for the enhanced sensitization of TMZ toward glioblastoma. Again, the individual combination of these drugs without nano-formulations such as liposomes has shown poor tumor regulation. From these observations, liposomes have played a vital role in contributing adequate therapeutic efficiency using this combination of drugs. The mice injected with these liposomes were able to avoid serious systemic adverse-drug reactions and exhibited the tumor-specific delivery of drugs by means of PEGylation. PEGylation not only induces the RES clearance and circulation times of liposome NPs but can also mitigate the identity of liposomes in the blood circulation. This improves the selective accumulation of liposome NPs in the brain tumors.

Substrate-mediated, liposome-based therapeutic platforms are also ideal for drug delivery because of their controlled drug release and excellent drug encapsulation. Hayward et al. developed hyaluronic acid (HA)-functionalized liposomes by embedding these in polyelectrolyte multilayer (PEM) through ionic bonding. This system sufficiently loaded hydrophilic (i.e., DOX) and hydrophobic components (i.e., cholesterol), and by utilizing PEM, sustainable delivery was achieved. For this, poly (sodium styrene sulfonate) (SPS) and poly-L-lysine (PLL) were employed as precursor substrates to form the multilayered films (around 4.5 bilayers). In this study, the SPS was utilized because of its extreme negative charge density and being a robust precursor for PEM preparation. Meanwhile, PLL was chosen for its potential features, such as outstanding positive charge density, better interaction capacity with HA, and suitable biocompatibility, etc. Further, these liposomes exhibited magnificent cellular uptake by metastatic breast cancer cells [66].

The integration of SPIONs and liposome NPs has achieved greater targeting ability against glioblastoma by thermal stimulation via alternating magnetic field (AMF) by means of SPIONs and a controlled drug-delivery response influenced by the liposomes. Shi et al. developed these dual-modalities by conjugating GBM-cell-specific peptides (P1NS) and antibodies (anti-GBM, i.e., tenascin-C (TN-C)) to the liposomes, co-loaded with SPIONs and DOX [67]. This affected the convenient passage through the BBB and facilitated higher amounts of cellular uptake by U-87 cells. The thermal stimuli were achieved from the SPIONs that induced mild hyperthermia, which targeted glioma cells by adjusting DOX-delivery specifically to glioblastoma without affecting the healthy cells. Moreover, TN-C was the highly expressed biomarker for glioblastoma, which involved the effective inhibition of cell proliferation along with DOX [68].

Generally, chemotherapeutic drugs target cell proliferation, cell division, or DNA synthesis for glioblastoma. Interestingly, the drugs dealing with the cell invasion of tumors are fewer [69]. This can be compensated by the usage of a few anti-depressants such as imipramine-blue (IB) and fluvoxamine. The IB-loaded liposomes can extend the survival rate of tumor-bearing rats by using DOX as a therapeutic drug [70]. This suggests that the integration of regular therapeutics with anti-invasive drugs can be an alternating strategy for drug-resistant GBM. In another study, the restructuring of the actin filaments was highly necessary for both the migration and invasion processes of tumor cells. To prevent the polymerization of actin, researchers screened the fluvoxamine, i.e., a selective serotonin reuptake inhibitor (SSRI), and enabled its penetration via the BBB without toxicity [71]. Moving to the screening method, fluorescence behavior (enhancement) of G-actin labeled with pyrene at the time of polymerization (a monomer) was noticed using fluvoxamine and other antidepressant drugs. Each drug was separately mixed with the reaction buffer comprising liposomes, the cytosol of mouse brain, GTP, and ATP. The fluvoxamine exerted sufficient prevention of actin polymerization. Fluvoxamine also successfully mitigated the cell migration as well as invasion of GBM and the stem cells of glioma through repressing focal adhesion kinase (FAK) and phosphoinositide-3-kinase (PI3K) pathways. These results suggest that fluvoxamine can be a promising therapeutic candidate against cell invasion in glioblastoma.

To achieve optimal theranostic applications, nanocarriers such as liposomes should guide high imaging and therapeutic activities by loading suitable agents. In this regard, Zhang et al. designed arsenic (As) and manganese (Mn) nanohybrid materials, which can target phosphatidylserine (PS) in the glioma cells [72]. The approach is based on the generation of magnetic susceptibility through the precipitate formation of arsenite (As^+3^)-Mn in the liposomes, which can be visualized as a T_2_-weighted dark contrast. However, after entering into the cells, the As-Mn complex will decompose at a low pH value and be released as As^+3^ and Mn^+2^ that provides a T_1_-weighted bright contrast.

More importantly, the surface of liposomes allows for the functionalization of polymers, antibodies, particular receptors, and enzymes, which can induce long circulation times in the bloodstream and aid in brain delivery [73]. Especially in glioblastoma, these functionalized liposomes can cross the BBB due to their suitable morphology and hydrophilicity. Recently, Ashrafzadeh and his coworkers designed liposomes by loading cisplatin and PEGylation, which showed maximum efficacy and declined toxicity against glioblastoma [74]. For this, they approached the transcytosis mechanism via selective targeting of the TfR receptors using liposomes. Several other reports are available to focus on these receptors due to their significant role in the progression of glioblastoma [75,76,77,78,79]. The expressions of the cell-surface glycoproteins (i.e., CD44) act as the leading targeting approaches against glioblastoma using liposome NPs. In this regard, the conjugation of the targeting ligand, namely hyaluronic acid, over the liposomes has demonstrated maximum cellular uptake in the glioma cells. Interestingly, no change in the size of observed HA-conjugated liposome NPs was observed. To compare the toxicity performance of these NPs, other healthy astrocytes and microglia were also employed, which did not show high toxicity, as in the case of glioblastoma cells. This proves the suitable efficacy of these NPs toward GBM cells by suppressing overexpressed CD44 markers in the GBM cells [80]. The receptor-targeted liposomes have shown an excellent result in the therapeutic understanding of glioma. For instance, drug (i.e., cisplatin)-loaded liposomes, targeted based on the VEGF (vascular endothelial growth factor) and VEGFR2 receptors, have shown magnificent accumulation in gliomas. Additionally, the conjugated liposomes exhibited high intracellular concentrations, which is beneficial against the growth of glioma cells. Their greater accumulation and cellular internalization can facilitate therapeutic activity [81]. Moreover, the integration of DOX-loaded liposomes with tumor necrosis factor (TNF) can progress the excellent apoptosis results compared to the DOX-loaded liposomes alone. This mainly results from the synergistic effect of tumor necrosis factor-related apoptosis-inducing ligand (TRAIL) and DOX-loaded liposomes by targeting the death receptor 5 (DR 5) in glioma cells [82].

### 3.4. Metal-Based Nanoparticles

These NPs are explored for treating glioblastoma using multiple approaches, such as hyperthermia and tumoral ablation. Hyperthermia involves the application of extreme temperatures into the tumoral tissues, which destructs the cell membranes and causes protein denaturation, DNA damage, and finally, apoptosis. Unfortunately, the burning of adjacent healthy tissues and the spreading of infections are the crucial demerits involved in this approach. However, to address these issues, metal NPs, gold nanomaterials especially, and gold nanorods are the best choices for their excellent tunable optical properties. Their surface plasmon resonance (SPR) features make the NPs convert the absorbed photon energy into heat, i.e., plasmonic photothermal therapy, which can tune the optical, thermal, and electrical properties of the NPs. This therapy can lead to the local disruption of the surrounding tumoral barrier that can enhance the delivery of drugs via the BBB. Conversely, the thermal ablation of tumoral tissues using elevated temperatures above 40 °C may lead to the generation of HSP proteins, which may also interact with the healthy tissues [83].

Gold nanorods with reasonably high biocompatibility and thorough functionalization can have the ability to interact with the tumor biomarkers. Choi et al. prepared PEGylated gold nanorods modified by arginylglycylaspartic acid (RGD) peptide, which could interact with αvβ3 integrins on the glioblastoma cells [84]. Even luminescent Ag NPs have also displayed robust plasmon features, including SPR absorption and surface-enhanced Raman scattering (SERS). The surface-functionalized (i.e., PEG) Ag NPs have previously bonded with αvβ3 integrins and demonstrated the binding efficiency of ligand and receptors against glioblastoma [85]. Tumor ablation using radiofrequency waves is fascinating, owing to their deep penetrative capacity into the severe tumoral tissues. However, it deals with a few drawbacks, such as non-selective heating with uneven destruction of tumoral tissues. To solve this, metal-based NPs have specifically targeted the tumoral tissue without affecting the surrounding tumors in a highly selective manner. For example, gold-based nanomaterials comprise excellent electrical and optical properties among other metallic materials [86].

### 3.5. Magnetic Nanoparticles

Magnetic nanoparticles (MNPs) are highly enticing nanomaterials with greater proportions of accumulation within the brain tumors, which can be conveniently monitored through MRI. For example, iron oxide NPs (e.g., Fe_3_O_4_ and Fe_2_O_3_) can have promising properties, such as biodegradability, non-toxicity, and superparamagnetic features, which facilitate tumor imaging and targeting applications [87,88]. These SPIONs are believed to be the best MRI contrasting agents for glioma imaging, and the suitable therapeutic candidates for magnetism-dependent hyperthermia. This behavior is attributed to their superparamagnetic nature, which allows them to exhibit extreme magnetic susceptibility; thereby, it can efficiently magnetize the NPs under the influence of external magnetic field [89]. Moreover, for effective brain permeability by the SPIONs, the physicochemical properties such as size, surface charges, and lipophilicity of the SPIONs need to be adjustable [90]. The characteristic functional groups such as amine (e.g., chitosan, polyethyleneimine), APTES (aminopropyl triethoxysilane), hydroxyl (e.g., PEG and polysaccharides), carboxyl (e.g., citrates, alginates, and polyacrylic acid) are popular in constructing functionalized iron oxide NPs. However, other functional moieties such as thiol, azide, and epoxy also play a crucial role for this purpose [91]. In this context, polymer-functionalized iron oxide NPs are extensively studied for their theranostic applications. This mainly results from their superior biological activities such as hemocompatibility and non-toxicity and their physicochemical properties such as facile surface-conjugation chemical reactions and the abundant presence of amine moieties [92,93,94,95]. Unfortunately, the delineation of boundaries for glioblastoma remains a struggling process before surgical operation procedures. To overcome and precisely identify the boundaries, the nanomaterial must have excellent MRI contrast imaging. To do so, Du and coworkers developed angiopep-2-cojungated PEGylated-ultrasmall SPIONs (USPIONs), which can successfully cross the BBB and specifically integrate with the lipoprotein receptor-based protein (LRP) of the GBM cells [96]. The T_1_-weighted positive contrasting ability of these USPIONs results from their ultra-small size (ca. 3.6 nm), leading to the potential MR imaging of glioblastoma cells before and after the surgical resection. Again, the PEGylation for the SPIONs has improved their colloidal stability and also enabled the covalent reaction between angiopep-2 and USPIONs.

Moving to the mechanism, SPIONs can rotate under the utilization of AMF, which results in the transformation of their kinetic energy into heat within the tumor tissues, finally leading to higher temperatures of around 46 °C. Likewise, SPIONs can affect tumors selectively via targeted apoptosis without affecting healthy cells [97]. Habra et al. functionalized the superparamagnetic iron oxide nanorods using a polymer, i.e., branched polyethyleneimine (BPEI), and loaded a dipeptide-based drug named “carnosine”. Interestingly, the smooth sponge-like matrix formed by the polymer could be able to prevent the rapid metabolism of carnosine. The mild hyperthermia (ca. 40 °C) produced by the magnetic oscillations of nanorods can help in the controlled release of carnosine against glioblastoma [98]. Here, we can praise the diagnostic performance of the SPIONs due to their extreme T_2_ relaxation that can improve the quality of the contrast of the images [99]. Hence, the magnetic NPs with highly qualified contrasting capacity can be utilized as excellent MRI diagnostic agents in investigating complex tumors.

Recently, lipid-based magnetic nanovectors were loaded using antibody-functionalized SPIONs for targeting GBM and endothelial cells of the BBB via higher expressions of TfR. In this study, researchers employed the dual therapy based on magnetic targeting and chemotherapy (i.e., TMZ) for targeting GBM cells. Additionally, the temperature-responsive lipophilic fluorescent label (i.e., dye: 1,1′-dioctadecyl-3,3,3′,3′-tetramethylindocarbocyanine perchlorate (DiI)) was used to investigate the temperatures within the GBM cells in response to the exposure of alternating magnetic field (AFM) [100]. Here, the used-lipid matrix can encapsulate SPIONs and allow the release of TMZ in a temperature-dependent manner and also provide magnificent stability for the SPIONs by inhibiting their aggregation.

Preferentially, ultra-small SPIONs are excellent alternatives as MRI contrasting agents with a very minimal renal toxicity and longer circulation times than gadolinium (Gd)-based contrasting agents in the brain tumors. These SPIONs show superior cellular uptake by the phagocytes (i.e., microglia) lying in the boundaries of tumors. Already, several reports have suggested that the combination of fluorescent molecules with MNPs can enhance tumor visualization and resection abilities. Previously, iron oxide nanomaterials modified with NRI fluorescent compounds such as Cy5.5 were beneficial for the detection of brain tumors with an excellent resection of tumors [101]. Thanks to the advancement of nanotechnology, greater targeting options such as antibodies, cytokines, chemotherapeutics, valuable peptides, proteins, and receptors have been contributed to the MNPs. In this regard, peptides and toxins are seemingly the potential conjugating candidates for MNPs in targeting brain tumors. For example, chlorotoxin is a toxin obtained from the venom of the scorpion and can interact with the enzyme, i.e., matrix metalloproteinase-2 (MMP-2), which can degrade the extracellular matrix (ECM) of tumors. Hence, chlorotoxin-modified MNPs act as dual MRI contrasting agents and potential tumor inhibitors in glioblastoma [102,103]. The other peptide, namely F3, can bind with the nucleolin of endothelial cells and can inhibit the proliferation of GBM cells [104]. Recently, thermotherapy by directly incorporating MNPs into the tumors has shown higher concentrations of MNPs and prolonged the supply of the magnetic field. This is a form of “localized hyperthermia”, which effectively prevents the infiltrating glioma from subsiding the healthy cells safely [105].

Radiofrequency (RF) hyperthermia is one of the most promising approaches for investigating brain tumors due to its high penetration capacity within a short time. For instance, Senturk designed an RF-based hyperthermia system (RF: 400 W and 13.56 MHz), which was integrated with magnetic polymeric NPs modified by a targeting ligand, i.e., glycine–arginine–glycine–aspartic acid–serine (GRGDS) peptide. These NPs enhance the therapeutic efficiency of the curcumin (a cytotoxic drug) with a 70% release within 72 h and with a heating temperature of about 43 °C in just 15 min. Interestingly, the GRGDS peptide has ameliorated the excellent cytotoxicity of these NPs against glioblastoma sixfold [106]. The combination of thermal and non-thermal properties of SPIONs and cell membrane respectively influence the cellular uptake and thereby potentiate the therapeutic efficiency of the curcumin in glioblastoma. The other polymer-based PEG/PEI/polysorbate 80 (Ps 80)-SPIONs were developed and loaded with DOX, which demonstrated superior suppression of glioma under the magnetic field. In this study, polymers helped extend blood circulation times and biocompatibility, whereas the DOX was used as a cytotoxic drug stimulated by SPIONs (i.e., magnetic field). Again, these polymer-functionalized SPIONs induced apoptosis in glioma cells via the caspase-3 pathway [107]. SPIONs are potential MRI contrasting candidates for diagnosing glioblastoma by targeting specific receptors expressed in glioblastoma. For this purpose, SPIONs were conjugated with TfR, which exhibited higher cellular uptake and internalization, and these SPIONs selectively penetrated the BBB and were adopted for MRI contrast agents [108].

### 3.6. Polymer-Based Nanoparticles

For many years, polymeric nanoparticles (PNPs) have been showing highly desirable properties such as maximum biodegradability, long-term stability, drug-loading capacity, and targeting ability, etc. These polymers include chitosan, cellulose, poly (lactic-co-glycolic acid) (PLGA), polyethylene glycol (PEG), alginates, and dendrimers. PNPs can cross the BBB via three mechanisms including (i) carrier-mediated transport (CMT), (ii) RMT, and (iii) adsorption-mediated transcytosis (AMT) [109]. More importantly, their self-assembling property can allow them to rapidly encapsulate DNA molecules, and again, non-toxicity and therapeutic efficacy can be greatly modulated by modifying with DNA-like biological entities. For instance, Cazares et al. developed highly biodegradable PNPs, which could deliver DNA into the brain tumor-initiating cells (BTICs). These non-viral NPs, i.e., poly (β-amino esters) (PBAEs), have shown outstanding stability with maximum efficiency toward the recurrence of glioblastoma [110].

The CMT mechanism deals with PNPs’ delivery into the BBB through carriers such as peptides, fatty acids, and amino acids, etc. For example, the expressions of transporters such as sodium (Na)-coupled carnitine transporter 2 (OCTN2) are crucial in the endothelium of glioma cells. Therefore, PNPs can target these transporters to attack the glioma cells. In this way, L-carnitine-conjugated PLGA NPs were synthesized and loaded with drugs such as paclitaxel (PTX) and Taxol to progress anti-tumoral activity against glioma cells [111]. Meanwhile, the RMT mechanism involves the passage of PNPs within the BBB utilizing the “expressions of the receptors”. Significantly, TfR-based receptors are promising targets for this mechanism due to their increased expressions. For example, TfR-conjugated magnetic silica-based PLGA NPs were loaded by DOX and PTX and showed sufficient internalization in the glioma tissues by crossing the BBB [112]. AMT is associated with the transcytosis process involved in the strong electrostatic interaction between positively charged ligands and negatively charged binding sites lying on the outer surfaces of cells [113]. For example, Lu and his coworkers designed cationic bovine serum albumin (BSA) modified PEG/PLA NPs and loaded them with a 6-coumarin for delivery into the brain [114]. Interestingly, cationic BSA NPs exerted a more magnificent internalization than the normal BSA NPs in the endothelial cells of the rat brain.

**Figure 4:**
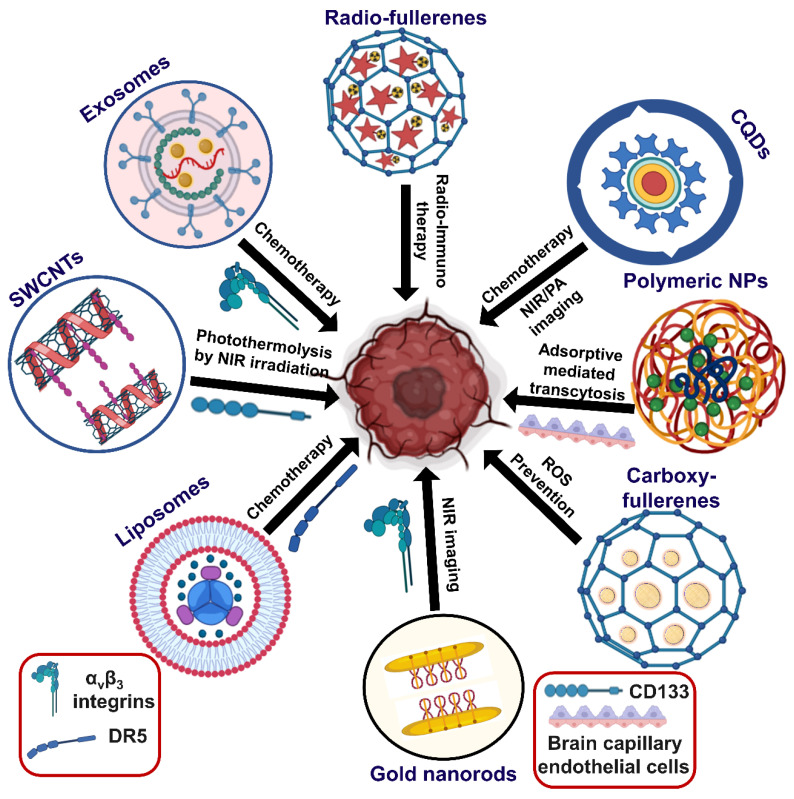
Recent theranostic and targeting approaches of nanomaterials against glioblastoma.

Nanotechnology has been contributing exciting opportunities, such as desired ligand functionalization and targeting abilities against tumor development. In addition, finding the optimal targeted receptor is crucial for the suitable drug design using nanocarriers. These emerging nanotheranostic approaches can exert broad potentials for understanding the inhibition capacity of glial tumors (Figure 4 and Table 1).

### 3.7. Clinical Trials-Related Studies of Nanomaterials for the Glioblastoma Treatment

Despite the prosperous therapeutic developments that have been advented against glioblastoma using nanomaterials, very limited progress in the conduction of clinical trials has been observed. So far, combinations of drugs such as TMZ and DOX loaded in the PEGylated liposomes have completed phase II clinical trials with the median overall survival of about 17.6 and 13.4 months in the two separate studies. In one study, after a year, progression-free survival (30.2%) was achieved after administering PEG-DOX, and continuous TMZ administration along with radiotherapy was reasonably tolerable [115]. However, the desired progress in the patients’ outcome was not achieved by these materials. In another study, the same combination showed a median overall survival of about 13.4 months without many therapeutic outcomes [116]. This suggested that it may have resulted from the drug resistance or inadequate therapeutic activity of the used drugs in clinical settings. Other cationic liposome-based viral vectors were utilized to deliver interleukin-12 through convection-enhanced delivery [117]. This required further biosafety profiles and comprehensive details for both progressive and recurrent glioblastoma. Though abundant studies have been conducted against glioblastoma using liposomes, they still require necessary vectors or drugs to validate promising biosafety measures in human clinical trials. Other types of nanomaterial such as magnetic iron-oxide nanoparticles have shown the prolonged overall survival against recurrent glioblastoma by employing thermotherapy and low dose-dependent radiation therapy [118]. Table 2 summarizes the details about clinical trials using nanomaterials toward the treatment of glioblastoma. Unfortunately, maintaining precise temperatures in tumors is a difficult task, which requires highly controllable and thermoresponsive magnetic NPs. Coming to the other nanomaterials, they are still in the budding stage for dealing with glioblastoma in terms of accuracy and biosafety. However, the emergence of nanohybrids with tunable physicochemical and biological activities will address these concerns in the near future.

## 4. Future Perspectives and Conclusions

Glioblastoma is a very severe and life-threatening pathological condition that cannot be treated by conventional therapies due to its heterogeneity and uncontrolled proliferation. In this context, nanomaterials can provide the opportunity for the efficient delivery of chemotherapeutic drugs by utilizing their physicochemical properties. Nanomaterials selectively display tunable optical and electrical properties. For example, Au and Ag NPs are promising materials to be regarded as “excellent theranostics” against glioblastoma. In the same manner, magnetic NPs are suitable for a continuous supply of magnetic fields, and thereby for heating the tumor regions. Currently, this form of hyperthermia caused by the AMF is highly attractive to researchers. Further, liposomal NPs safely cross and deliver the different drugs into the brain through the BBB. Nevertheless, highly biodegradable polymeric NPs pass the BBB via different mechanisms into the tumor sites and increase the concentrations of drugs with high specificity. Excitingly, carbon-based nanomaterials are promising theranostics with excellent optical properties, i.e., multicolored fluorescence and functionalization abilities of ligand/chemotherapeutics. Hopefully, these developments in nanomaterials can be explored for the diagnosis and treatment of glioblastoma.

## Figures and Tables

**Table 1:** Nanotechnology-based theranostic approaches to glioblastoma.

Nanomaterials	Ligands or Precursors	Targeted Drugs or Radiation Source	Expressed Receptors or Markers	Approaches	Ref.
ESC-derived exosomes	c(RGDyK)	PTX	α_v_β_3_integrins	Chemotherapy	[41]
Carboxyfullerenes (C_70_)	Malonic acid	-	-	ROS prevention	[46]
Radio-fullerenes (C_80_)	IL-13 peptide	β-emitters		Radio-immunotherapy (RIT)	[48]
Magnetic nano GO SPIONs	Poly lactic-*co*-glycolic acid (PLGA)	5-Iodo-2-deoxyuridine (IUdR)	Bax/Bcl-2	MRI/radiotherapy	[51]
CQDs	TAAQ and CA	DOX, hydroxycamptothecin, topotecan hydrochloride (TPTC)	-	Chemotherapy and NIR/PAimaging	[54]
SWCNTs	CD133 MoAb	TMZ	CD133	Photothermolysis by NIR laser irradiation	[58]
LNPs	Hyaluronic acid	DOX	CD44	Chemotherapy	[80]
Liposomes	TNF	DOX/ TRAIL	DR5	Chemotherapy	[82]
Gold nanorods	PEG and RGD peptide	NIR	α_v_β_3_integrins	NIR imaging	[84]
Ag NPs	PEG and c-RGD peptide	Fluorescence and SERS	α_v_β_3_integrins	Fluorescence and SERS	[85]
SPIONs	PEG/PEI/polysorbate 80	DOX	Caspase-3	Chemotherapy	[107]
PNPs	BSA/PEG/PLA	6-coumarin	BCECs	AMT	[114]

**Table 2:** Clinical trial-related developments of the nanotechnology-based materials for glioblastoma.

Nanomaterials	Disease	Loaded Drugs/ Therapy	Phase	Results	Ref
PEGylated liposomes	Glioblastoma	DOX and TMZ	Phase II	Median overall survival (17.6 months)	[115]
PEGylated liposomes	Glioblastoma	DOX and TMZ	Phase II	Median overall survival (13.4 months)	[116]
Cationic liposomes	Recurrent glioblastoma	Interleukin-12	Phase I, II	Convection-enhanced delivery through virus vector	[117]
Magnetic iron-oxide nanoparticles	Recurrent glioblastoma	Thermotherapy and lower radiotherapy dose (30 Gy)	Phase II	Effective and prolonged overall survival	[118]

## Abbreviations

BBB	blood–brain barrier
NIR	near-infrared
PA	photoacoustic
EPR	enhanced permeability and retention
PDT	photodynamic therapy
GBM	glioblastoma multiforme
AuNPs	gold nanoparticles
AgNPs	silver nanoparticles
PEG	polyethylene glycol
ROS	reactive oxygen species
PTT	photothermal therapy
AMF	alternating magnetic field
DR5	death receptor 5
SERS	surface-enhanced Raman scattering
PLGA	poly (lactic-co-glycolic acid)
CMT	carrier-mediated transport
RMT	receptor-mediated transcytosis
AMT	adsorption-mediated transcytosis
BTICs	brain tumor-initiating cells
PBAEs	poly (β-amino esters)
BSA	bovine serum albumin
